# Identification of diagnostic markers for moyamoya disease by combining bulk RNA-sequencing analysis and machine learning

**DOI:** 10.1038/s41598-024-56367-w

**Published:** 2024-03-11

**Authors:** Yifan Xu, Bing Chen, Zhongxiang Guo, Cheng Chen, Chao Wang, Han Zhou, Chonghui Zhang, Yugong Feng

**Affiliations:** Department of Neurosurgery, The Affiliated Hospital of Qingdao University, 16 Jiang Su Road, Qingdao City, 266000 China

**Keywords:** Moyamoya disease, Bulk RNA-sequencing, Machine learning, Immune infiltration, Diagnostic biomarkers, Biomarkers, Neurology

## Abstract

Moyamoya disease (MMD) remains a chronic progressive cerebrovascular disease with unknown etiology. A growing number of reports describe the development of MMD relevant to infection or autoimmune diseases. Identifying biomarkers of MMD is to understand the pathogenesis and development of novel targeted therapy and may be the key to improving the patient’s outcome. Here, we analyzed gene expression from two GEO databases. To identify the MMD biomarkers, the weighted gene co-expression network analysis (WGCNA) and the differential expression analyses were conducted to identify 266 key genes. The KEGG and GO analyses were then performed to construct the protein interaction (PPI) network. The three machine-learning algorithms of support vector machine-recursive feature elimination (SVM-RFE), random forest and least absolute shrinkage and selection operator (LASSO) were used to analyze the key genes and take intersection to construct MMD diagnosis based on the four core genes found (ACAN, FREM1, TOP2A and UCHL1), with highly accurate AUCs of 0.805, 0.903, 0.815, 0.826. Gene enrichment analysis illustrated that the MMD samples revealed quite a few differences in pathways like one carbon pool by folate, aminoacyl-tRNA biosynthesis, fat digestion and absorption and fructose and mannose metabolism. In addition, the immune infiltration profile demonstrated that ACAN expression was associated with mast cells resting, FREM1 expression was associated with T cells CD4 naive, TOP2A expression was associated with B cells memory, UCHL1 expression was associated with mast cells activated. Ultimately, the four key genes were verified by qPCR. Taken together, our study analyzed the diagnostic biomarkers and immune infiltration characteristics of MMD, which may shed light on the potential intervention targets of moyamoya disease patients

## Introduction

MMD is a rare chronic occlusive cerebrovascular disease characterized by reduced cerebral blood flow due to stenosis or occlusion of the cranial carotid arteries, often secondary to abnormal formation of the skull base vascular network^1^. In East Asia, the incidence of moyamoya disease is much higher than in other areas^2,3^. In a national survey in Japan, among 7700 patients surveyed, the ratio of female to male patients was 1.8, and the peak age of onset of patients was described as 10 to 14 years for females and 20 to 24 years for males^4^. Furthermore, studies have depicted that the hemodynamics of patients with moyamoya disease has also changed, the dilated and fragile moyamoya membrane blood vessels often rupture and cause intracranial hemorrhage. Consequently, searching novel biomarkers related to MMD and improving the accuracy of MMD prediction is key to improving MMD prevention and management.

Little is known about the etiology and pathogenesis of MMD, recent studies have shown that it may be influenced by genetic, immune response, inflammation^5,6^. It has been confirmed that the ring finger protein 213 (RNF213) is the most crucial susceptibility gene of MMD^5,7^. A few MMD patients, however, did not have RNF213 mutation, which may be related to innate angiogenesis. Many research findings revealed that the increase or abnormal activity of some growth factors such as vascular endothelial growth factor (VEGF), basic fibroblast growth factor (bFGF), hepatocyte growth factor (HGF), can promote intimal hyperplasia and smooth muscle cell (SMC) migration in vessels^8–10^. Additionally, it is reported that many autoimmune diseases are related to moyamoya disease, such as systemic lupus erythematosus, graves disease, antiphospholipid antibody syndrome and HLA class I or II allele abnormalities^11–13^. Previous studies suggested that IgG was deposited in the damaged inner elastic layer, which promoted S100A4 migration to the intima of blood vessels, leading to lumen stenosis and compensatory proliferation of small blood vessels, indicating that immune-related factors may be involved in the functional and morphological changes of smooth muscle cells^14^. Fujimura et al. Found that the concentrations of sCD163 and CXCL5 in serum were abnormal and concluded that M2 macrophages might participate in the pathogenesis of MMD by increasing their autoimmune activity^15^. Kang et al. found that the increase of IL-1β level secreted by macrophages can activate the proliferation of macrophages, endothelial cells and smc, thus leading to the increase of vascular permeability and endothelial dysfunction^8^. These studies have proved that the abnormal immune system may exert a key part in the MMD formation.

In this study, GSE157628 and GSE141024 datasets were obtained in GEO database, the WGCNA algorithm was used to investigate gene variants and explore the coexpression network most closely related to MMD. Before this, nevertheless, there has never been any investigation using machine learning, this study is the first application of machine learning to determine the characteristic genes of MMD immune-related genes. Here, we apply three machine learning algorithms, random forest, SVM-RFE and LASSO, and to predict biomarkers, to predict the MMD progress. All the work we do is aimed at finding emerging and accurate biomarkers and clinical intervention targets that can be used in the diagnosis and treatment of MMD.

## Methods

### Data processing and download

**Figure 1:**
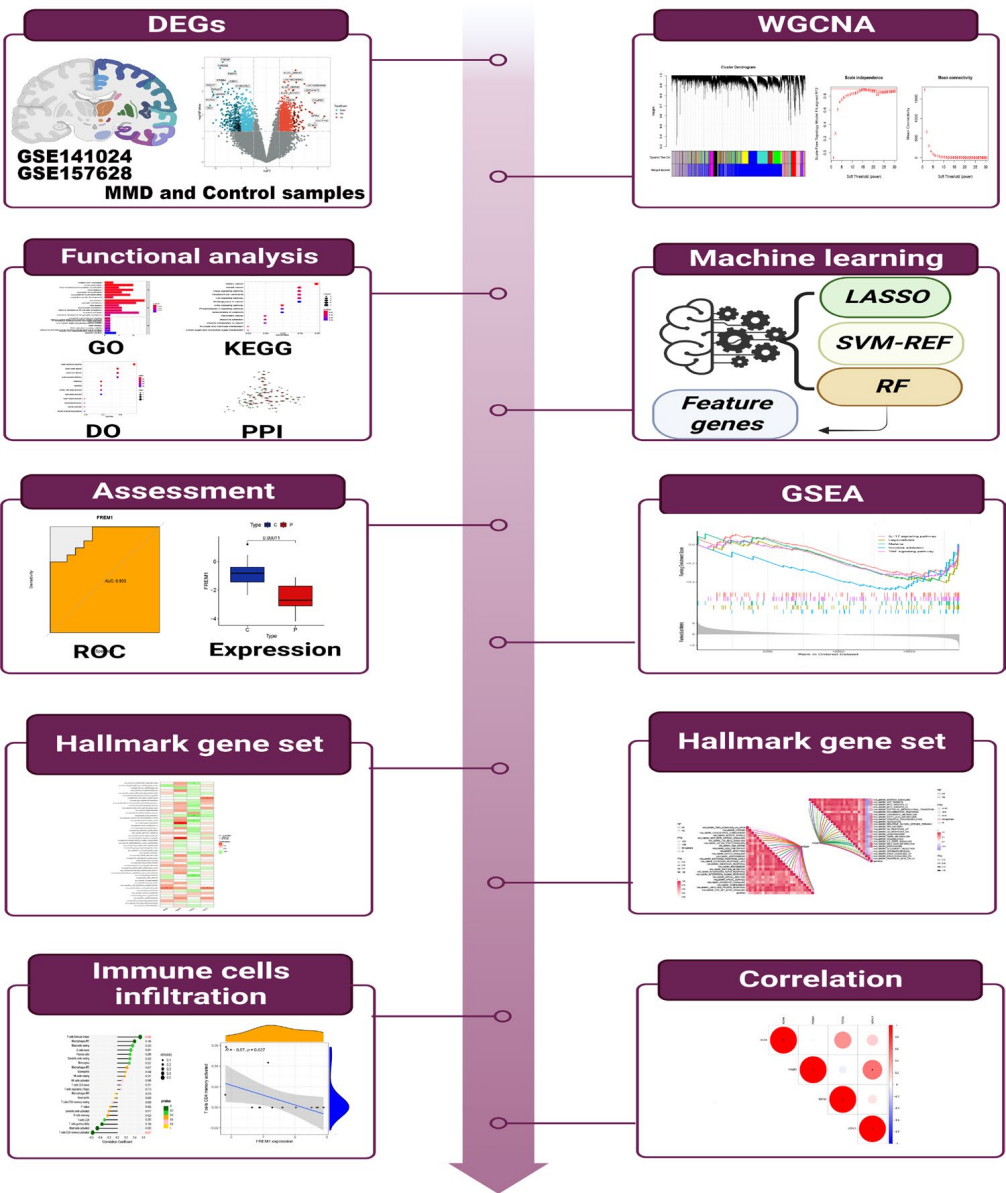
The flowchart of analysis procedure.

GSE157628 and GSE141024 were obtained from the GEO (https://www.ncbi.nlm.nih.gov/gds) database, details of the two datasets are found in Supplementary Table S1. And some of their clinical characteristics are found in Supplementary Tables S2, S3. The raw data were processed and normalized to use the "limma" (version 3.46.0) R package, including the probe ID transformation and calculation of gene expression. To eliminate batch effects from the dataset, we employed the "sva" R package. The workflow of this investigation is provided in Fig. 1.

### Differentially expressed gene (DEG) analysis

The aim of this study was to conduct a differential expression analysis in order to investigate the disparities between normal patients and those diagnosed with moyamoya disease. DEG analysis was performed using the "limma" R package under the conditions of p < 0.05 and |log2FC|≥ 0.5. The genes are categorized as up-regulated or down-regulated based on whether their log 2FC value exceeds 0.5 or falls below − 0.5. In order to enhance the visualization of these differentially expressed genes (DEGs), R software is utilized to generate heat maps and volcano plots. Heat maps are constructed using the pheatmap R package.

### Enhancement of functionality

The data is assessed through functional enrichment analysis to validate the potential target's putative function. Gene Ontology (GO), the Kyoto Encyclopedia of Genes and Genomes (KEGG)^16–18^ and Disease ontology (DO) were used to estimate functional enrichment by the "GOplot”, “cluster profiler” and “DOSE” packages in R. Statistical significance was set at p < 0.05. The PPI networks can be utilized for generating gene function predictions and identifying genes with comparable effects. Network integration algorithms employ various bioinformatics methods such as physical interaction, co-expression, co-localization, gene enrichment analysis, genetic interaction, and site prediction. The construction of PPI networks involved the utilization of a string database (https://string-db.org/), and MMD-related immune genes were selected based on confidence levels exceeding 0.4.

### Weighted co-expression network analysis (WGCNA)

The WGCNA distinguishes the gene co-expression network into several highly related characteristic modules and can associate the modules with specific clinical features, find key genes, help identify latent mechanisms involved in specific biological processes and seek candidate biomarkers^19^. Pearson correlation analysis is used to generate the similarity matrix between key genes, then the adjacency matrix is calculated, and the topological overlap matrix (TOM) is constructed and using (1-TOM) to describe the dissimilarity between genes to identify hierarchical clustering nodes and modules. Subsequently, the highly similar modules are determined by cluster analysis. The coexpression modules that meet the conditions (deepSplit = 2, height = 0.25, minModuleSize = 50) were identified by the DynamicTreeCut function.

### Identification of potential key genes

In this study, three machine learning algorithms, SVM-RFE, random forest and LASSO, were used to isolate characteristic genes. SVM-RFE is a machine learning algorithm based on the maximum interval theorem of SVM. It adopts the principle of minimizing structural risks and minimizing empirical errors, to strengthen the learning performance^20^. The SVM module was developed by the “e1071” package. LASSO regression is characterized by fitting generalized linear model and screening variables,which analysis was realized by glmnet software package with tenfold cross-verification through a turning/penalty parameter^21^. RandomForest is used to rank genes. Ultimately, we combine three machine learning modes to further screen the most significant feature genes. Receiver Operating Characteristic (ROC) curve and area under ROC (AUC) were used to evaluate the diagnostic value of biomarkers.

### Gene set enrichment analysis (GSEA)

GSEA was utilized to determine the biological significance of obtained feature genes, which was referenced “c2.cp.kegg.v11.0.symbols” gene sets (http://software.broadinstitute.org/gsea/msigdb) at a criterion of FDR < 0.05. Besides, correlations between optimal feature gene expression levels were calculated using Pearson correlation analysis.

### Immune infiltration analysis

The immune infiltration level of each sample was analyzed by CIBERSORT analysis technique^22,23^. The normalized gene expression matrix was uploaded to the CIBERSORT server (https://cibersort.stanford.edu/). Absolute and relative modes were applied while disabling quantile normalization. A total of 1000 permutations were conducted for statistical testing. The resulting output provides the percentage distribution of immune cell types across all samples, ensuring that the sum of immune cell ratios for each sample equals 1. The Wilcoxon rank-sum test was used to evaluate the differences in immune cell proportions and p < 0.05 was considered statistically significant.

### Quantitative real-time PCR

We extracted total RNA from tissues using AxyPrep Multi-source Total RNA Micropreparation Kit (Thermo Scientific, K0731). The total RNA of lμg was also used for cDNA synthesis by using a reverse transcription kit (Thermo Scientific, K16225). Real-time quantitative PCR was performed using THUNDERBIRD SYBR qPCR Mix (Toyo Spun, Shanghai, China) on anABIPRISM 7500HT instrument (Applied Biosystems) to detect the expression of mRNA. Taking the relative ratio of target gene to GAPDH as its expression, the relative ratio was calculated by 2−ΔΔCt method. Targeted gene primer sequences were as follows: ACAN, CTCACCATCCCCTGCTATTTCAT (forward), ACACGGCTCCACTTGATTCTT (reverse); FREM1, CCTTCCCAACGAAGTCAAGTATG (forward), CACCTCCAGCACATTGTTACTC (reverse); TOP2A, AGGATTCTGCTAGTCCACGATAC (forward), CACCATGGGAATAATAGGAATGTACC (reverse); UCHL1, GAGCTGAAGGGACAAGAAGTTAG (forward) GGCCACTGCGTGAATAAGTC (reverse).

### Statistical analysis

All statistical tests were carried out by R software version 4.1.3. The Kruskal‒Wallis test was used for variable comparison between multiple groups. Wilcoxon rank-sum test was utilized for analyzing the difference between the two groups. The correlation among the variables was determined to use Pearson’s or Spearman’s correlation test.All statistical p-values were two-sided, and p < 0.05 was regarded as statistical significance.

### Ethics statement

Written informed consent was obtained from the individual(s) for the publication of any potentially identififiable images or data included in this article. On behalf of all authors, I guarantee that all experiments involving human tissue samples were conducted in accordance with relevant guidelines and regulations, and that all experimental protocols were approved by the Ethics Committee of the Affiliated Hospital of Qingdao University.

## Results

### DEG screening and data preprocessing

**Figure 2:**
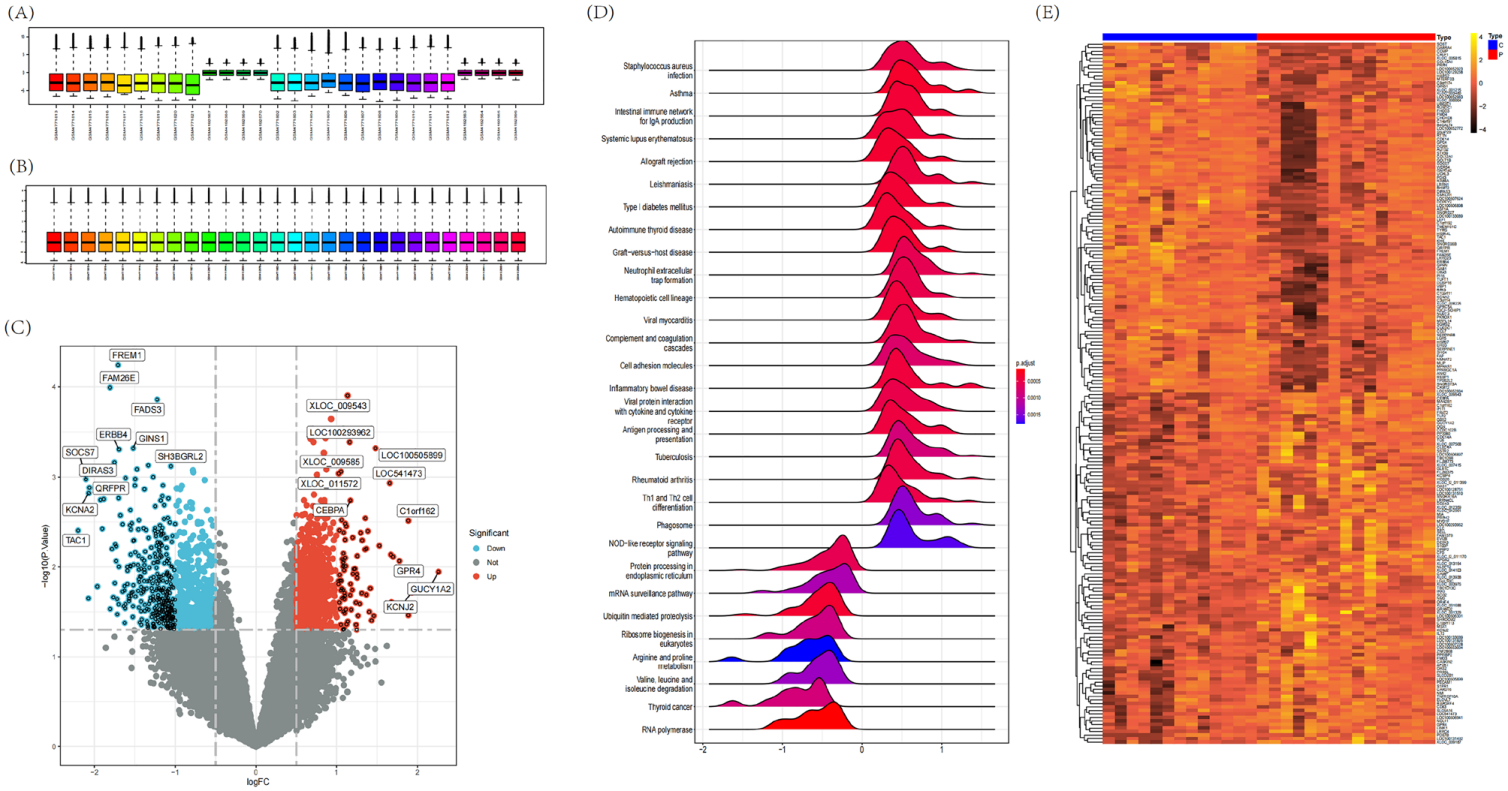
Normalization of all samples and DEG analysis. (**A**) Sample expression box diagram before normalization. (**B**) Sample expression box diagram after normalization. (**C**) The volcano map shows the identified DEGs, with 83 genes up-regulated and 331 genes down-regulated. (**D**) Ridgeline plot of DEGs. (**E**) Heatmap of DEGs. The first column displays the group information, while each row represents a single gene and each column presents data from a specific sample. Up-regulated genes are depicted in a vibrant color, whereas down-regulated genes are portrayed in a darker shade.

We studied the role of immune-related genes in the progress of moyamoya disease by combining sample expression profiles from GSE157628 and GSE141024 cohorts. We used PCA to verify the consistency of the sample distribution prior to and after correction. Figure S1A displays the scatter distribution of the two datasets before batch effect removal, while Fig. S1B depicts the scatter distribution after correction, indicating the successful removal of the confounding factors from the rectified samples. Figure 2A,B shows the normalization and DEG analysis of all samples, rows represent samples, and columns represent gene expression values in samples. The volcano plot shows the recognized DEGs, with 83 genes up-regulated and 331 genes down-regulated (Fig. 2C, Supplementary Table S4). Ridgeline plot indicated changes in various biological functions and processes in MMD (Fig. 2D). The heat map depicted in Fig. 2E demonstrates DEGs.

### Screening of feature modules by WGCNA

**Figure 3:**
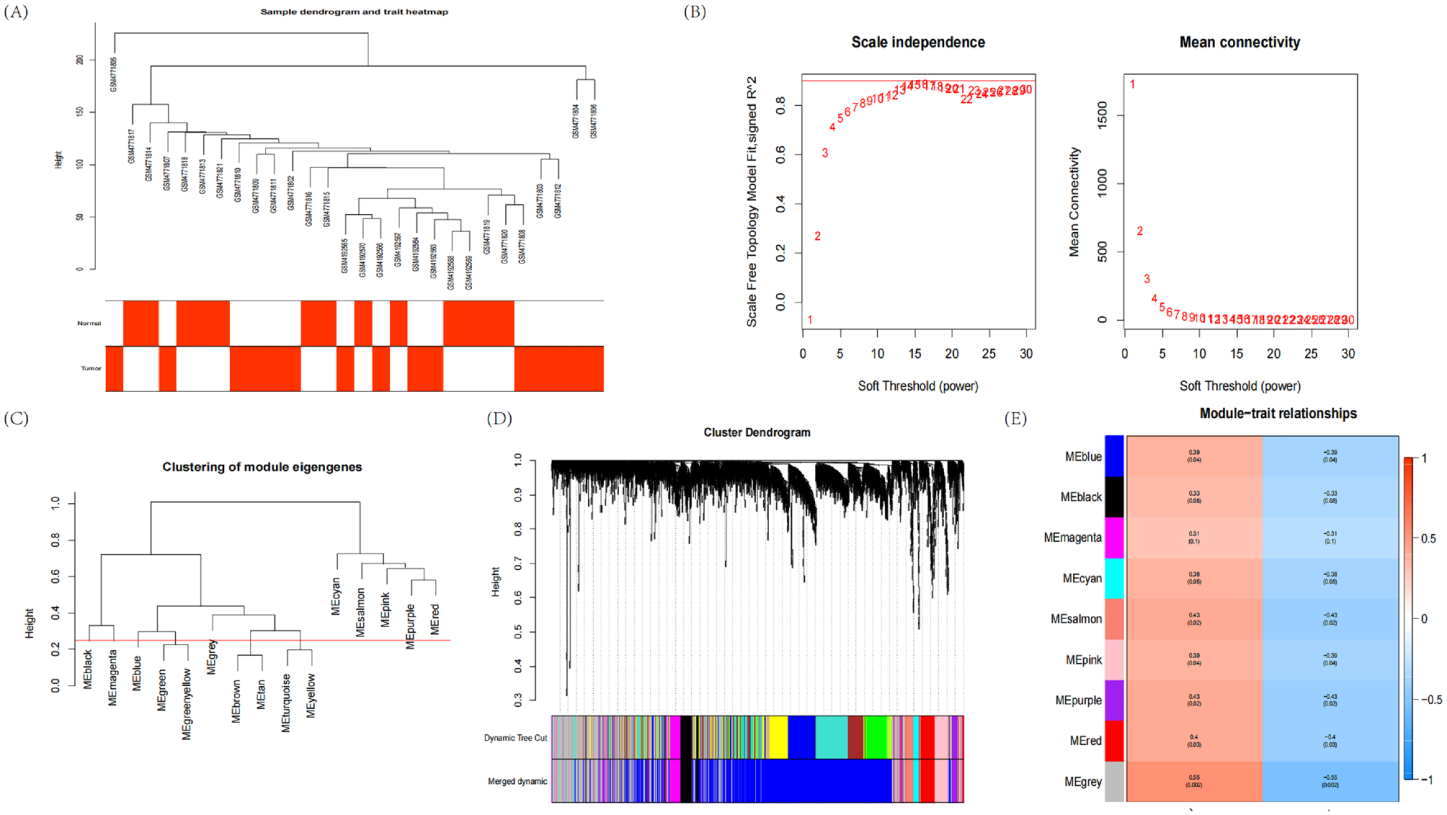
Construction of WGCNA co–expression network. (**A**) Sample clustering dendrogram with tree leaves corresponding to individual samples. (**B**) Soft threshold b = 6 and scale–free topological fit index (R2). (**C**) Clustered dendrogram were cut at a height of 0.25 to detect and combine similar modules. (**D**) The cluster dendrogram of the genes with median absolute deviation in the top 25%. Each branch in the figure represents one gene, and every color below represents one co-expression module. (**E**) Heat map of module–trait correlations. Red represents positive correlations and blue represents negative correlations.

**Figure 4:**
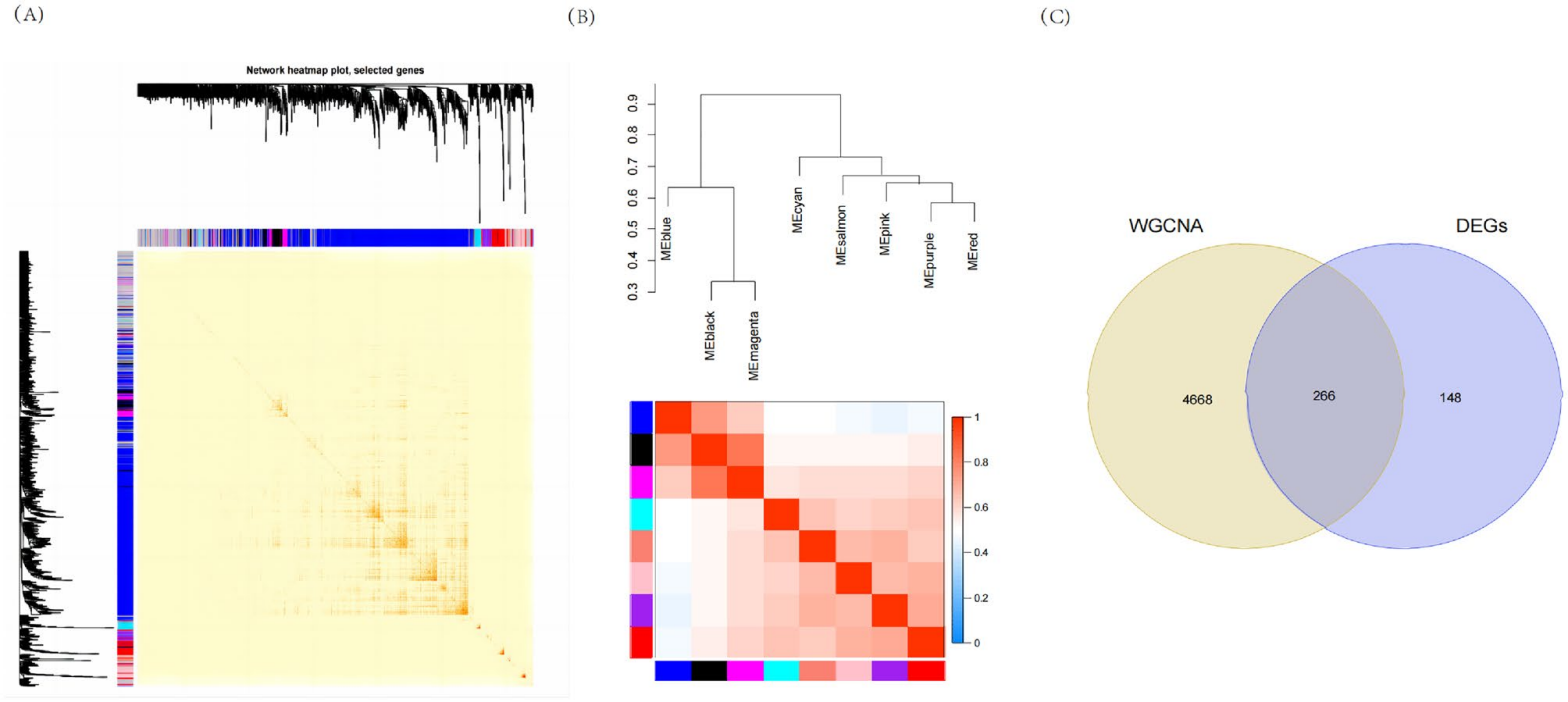
Construction of WGCNA co–expression network. (**A**) Clustering dendrogram of module feature genes. (**B**) Collinear heat map of module feature genes. Red color indicates a high correlation, blue color indicates opposite results. (**C**) Venn diagram of key module genes versus DEGs.

The samples in GSE157628 and GSE141024 datasets were clustered, and the gene expression matrix containing 7001 genes with a standard deviation greater than zero was obtained. To eliminate abnormal samples, we set a threshold (Fig. 3A). For another, establish a scale-free network through the "pickSoftThreshold" of the "WGCNA" package, set the power parameter range to 1–30 and the soft threshold to 6, as shown in Figs. 3B,C. Eight modules are determined based on average hierarchical clustering and dynamic tree cutting (Fig. 3D). Figure 3E shows the frontal correlations between clinical features and ME value. The reliability of module interaction is proved by transcription correlation analysis within the module, which indicates that there is no substantial connection between modules (Fig. 4A). The results of the independence test among the modules show that there is no correlation between each module (Fig. 4B).

### Functional enrichment analysis

**Figure 5:**
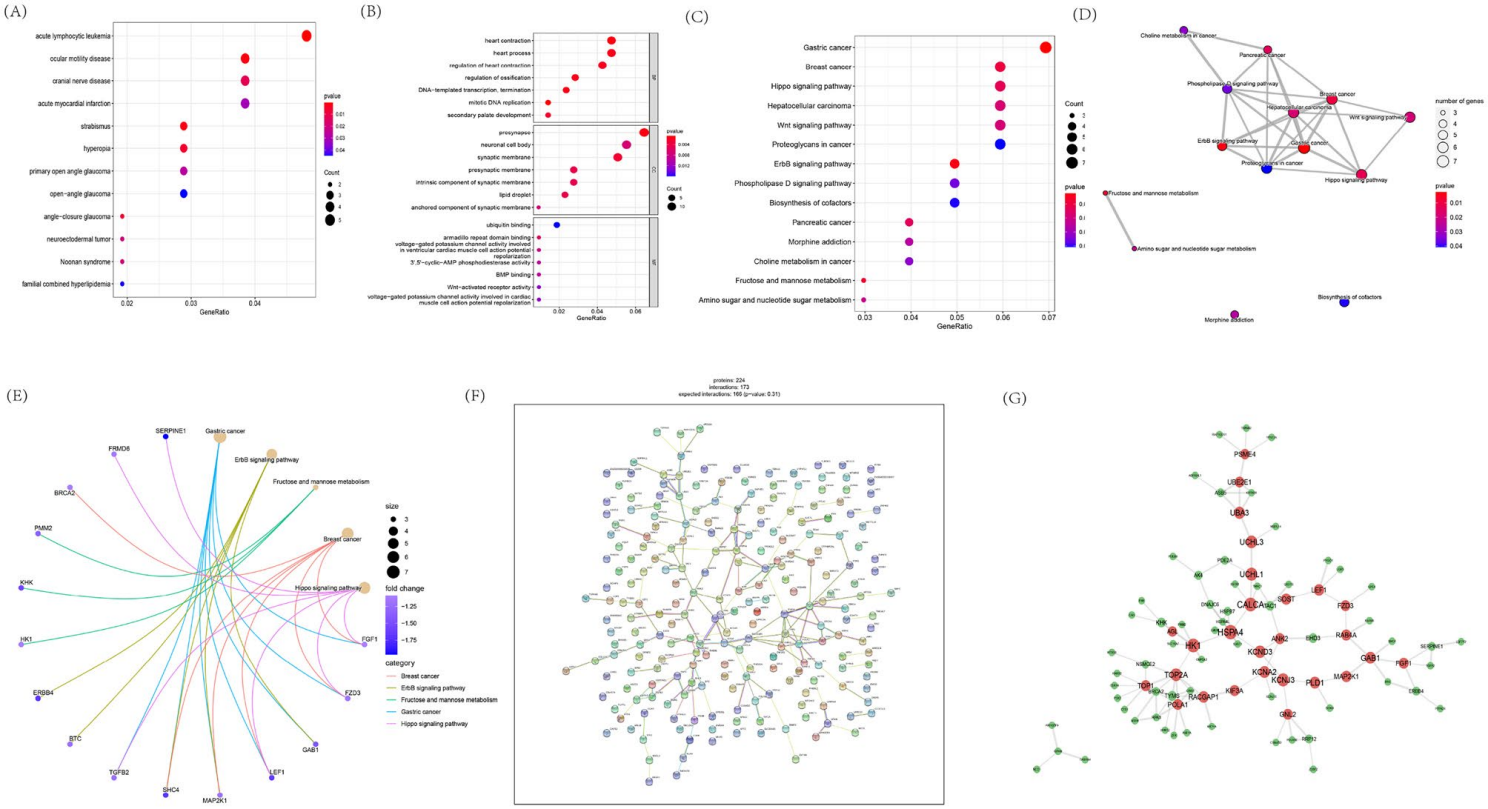
Analyses of functional enrichment of DEGs and PPI network. (**A**) DO analysis of co-expressed genes. (**B**) GO analysis of co-expressed genes. (**C**–**E**) KEGG analysis of co-expressed genes. (**F**) The PPI network of feature genes. (**G**) The co-expression network showing the correlation intensity of hub genes from overlapping candidate genes.

Venn plot illustrated that there were 266 common genes between DEGs and WGCNA module genes (Fig. 4C, Supplementary Table S5). The consequences of DO analysis illustrates that these common genes are relevant to acute lymphocytic leukemia, ocular motility disease, cranial nerve disease and acute myocardial infarction (Fig. 5A). Exploring MMD-related signal pathways by applying GO analysis, which mainly were divided into three categories: cell components (CC), biological processes (BP) and molecular functions (MF). GO enrichment analysis revealed that those 266 common genes were closely related to BPs such as heart contraction, heart process; CCs such as presynapse, neuronal cell body; and MFs such as ubiquitin binding and armadillo repeat domain binding (Fig. 5B). It is also worth noting that, several common cancers, such as gastric cancer, breast cancer, and hepatocellular carcinoma, were enriched by KEGG analysis, which means that MMD may have a similar or identical molecular mechanism to cancer progression. In addition, we also noticed some common pathways, such as Hippo, Wnt, ErbB signaling pathway, etc. (Fig. 5C–E). Meanwhile, we established a hub module from PPI network, including key atherosclerotic plaque progression and immune-related genes (Fig. 5F,G). Statistical significance was set at p < 0.05.

### Verification of diagnostic marker genes

**Figure 6:**
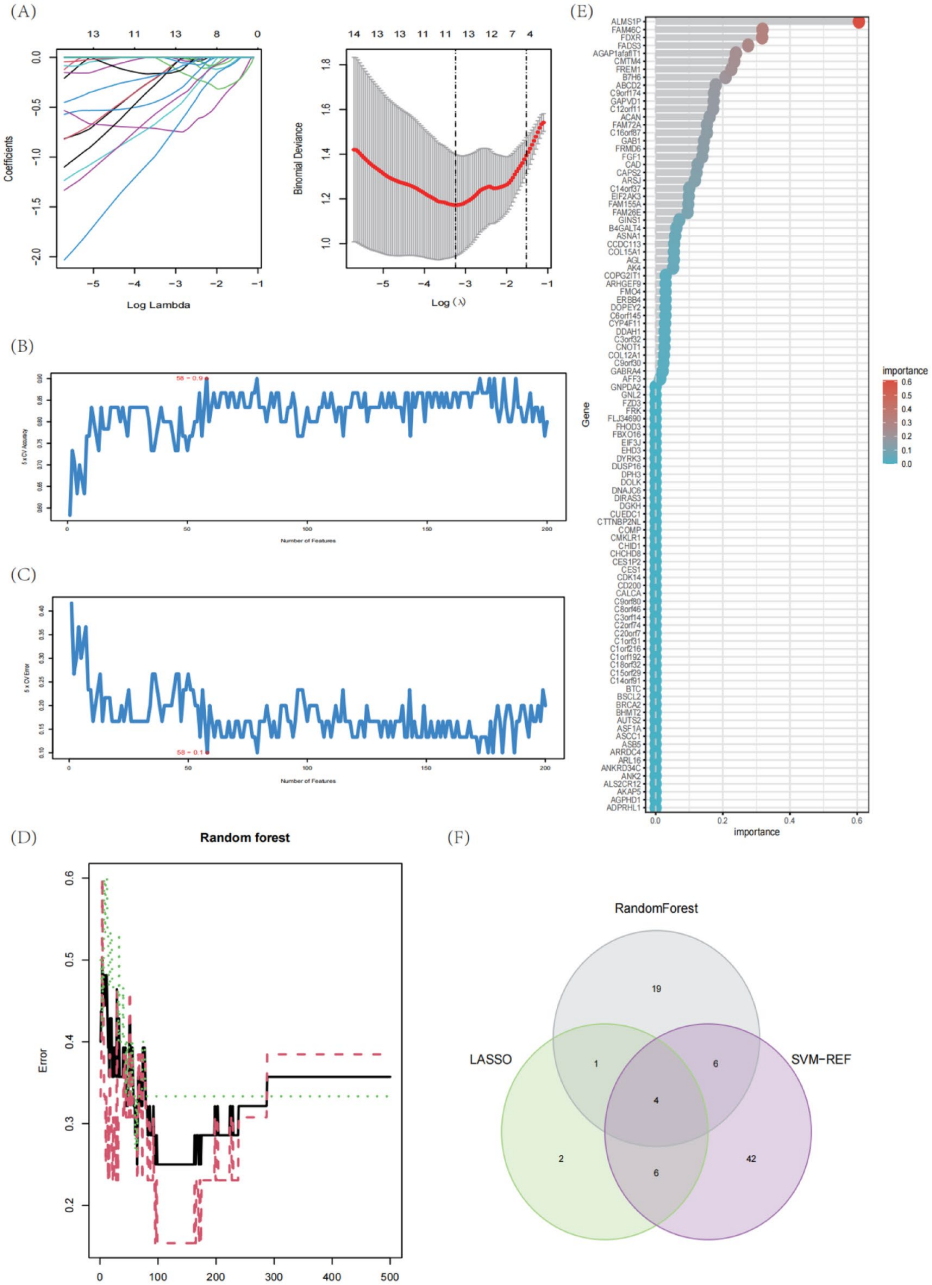
Diagnostic marker genes selection. (**A**) The performance in of ten-time cross-verification for tuning parameter in selection LASSO. (**B**,**C**) Biomarker signature gene expression validation by support vector machine recursive feature elimination (SVM–RFE) algorithm selection. (**D**) randomForest error rate versus the number of classification trees. (**E**) The top 50 relatively important genes. (**F**) Venn plot shows the key genes screened by three machine learning methods.

**Figure 7:**
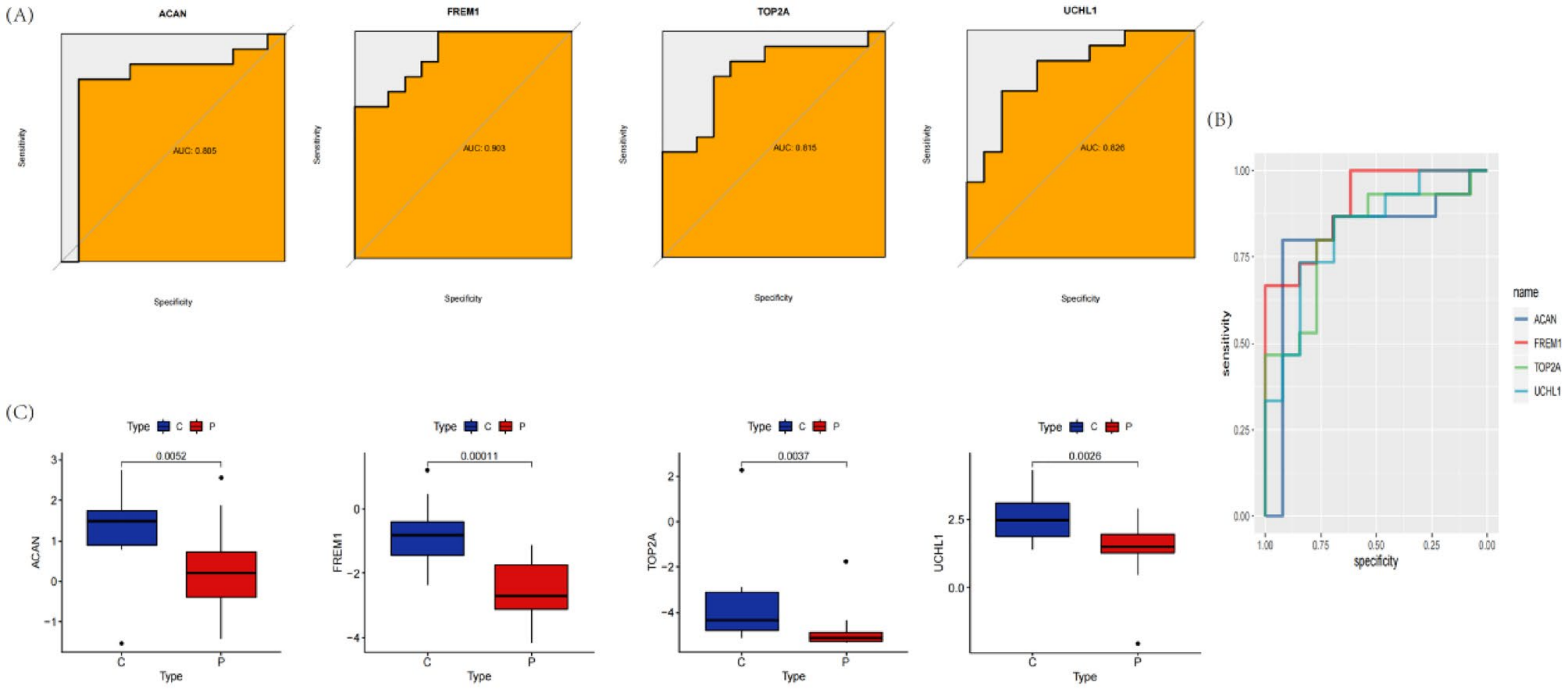
MMD diagnostic value and characterized gene expression validation. (**A**,**B**) ROC curves of the feature genes. (**C**) Diagnostic marker gene expression in GSE157628 and GSE141024 datasets.

We utilize machine learning algorithm to choose the foremost features to screen hub genes with the most diagnostic value. Thirteen key biomarkers were identified from deg by LASSO logistic regression (Fig. 6A). Fifty-eight genes were obtained as diagnostic markers by SVM-RFE algorithm (Fig. 6B,C). The RF algorithm determines 30 genes as key indexes (Fig. 6D,E). By screening overlapping genes from LASSO, random forest and SVM-RFE, we eventually got 4 shared hub genes, which are considered to have the greatest diagnostic value, with ACAN, FREM1, TOP2A and UCHL1 respectively (Fig. 6F). To further verify the diagnostic and prognostic efficacy of each shared central gene, we used ROC curve and AUC values for evaluation (Fig. 7A,B). For confirming the previous findings, we validated the expression differences of these four genes between samples of different states in two downloaded datasets and observed that ACAN, FREM1, TOP2A and UCHL1 was significantly downregulated in MMD samples (Fig. 7C).

### Identification of the function of four diagnostic marker Genesc

**Figure 8:**
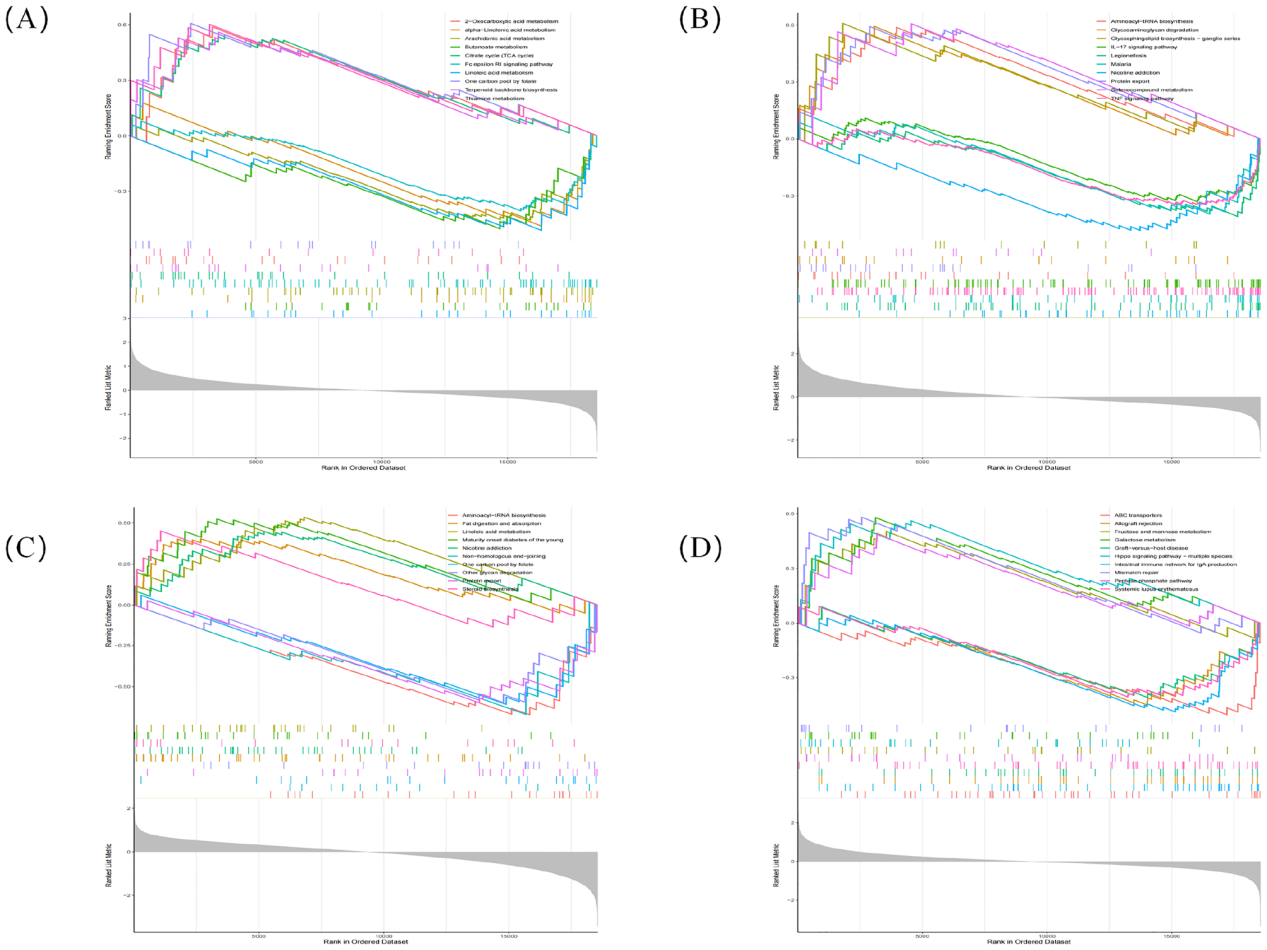
GSEA identifies signaling pathways involved in the diagnostic marker genes. (**A**) GSEA analysis of ACAN gene. (**B**) GSEA analysis of FREM1 gene. (**C**) GSEA analysis of TOP2A gene. (**D**) GSEA analysis of UCHL1 gene.

We use GSEA to classify MMD tissues into two categories according to the median expression of each single signature genes. According to ACAN, In the highly expressed subgroup, one carbon pool by folate, terpenoid backbone biosynthesis, thiamine metabolism, citrate cycle (TCA cycle), 2-oxocarboxylic acid metabolism were significantly enriched, whereas alpha-linolenic acid metabolism, arachidonic acid metabolism, butanoate metabolism, fc epsilon RI signaling pathway, linoleic acid metabolism were significantly enriched in the low ACAN subgroup (Fig. 8A). As for FREM1, aminoacyl-tRNA biosynthesis, glycosaminoglycan degradation and glycosphingolipid biosynthesis-ganglio series and selenocompound metabolism protein export were significantly enriched in the high FREM1subgroup, whereas IL-17 signaling pathway, legionellosis, malaria, nicotine addiction, TNF signaling pathway were significantly enriched in the low FREM1 subgroup (Fig. 8B). In the high TOP2A subgroup, fat digestion and absorption, linolenic acid metabolism, maturity onset diabete of the young, the nicotine addiction and steroid biosynthesis were significantly enriched, whereas were significantly Aminoacyl-tRNA biosynthesis non-homologous end-joining, one carbon pool by folate, other glycan degradation and Protein export enriched in the low TOP2A subgroup (Fig. 8C). In the high UCHL1 subgroup, fructose and mannose metabolism, galactose metabolism, hippo signaling pathway-multiple species and pentose phosphate pathway were significantly enriched whereas systemic lupus erythematosus, ABC transporters allograft rejection, Intestinal immune network for IgA production, Graft-versus-host disease were significantly enriched in the low UCHL1 subgroup (Fig. 8D). The Neo4j browser was ultimately utilized for conducting additional GSEA analysis, which yielded several enriched pathways: matrix metalloproteinases (MMPs), collagen degradation, hyaluronic acid binding, extracellular matrix (ECM) proteoglycans and epinephrine binding (Supplementary Table S6). Overall, the GSEA enrichment differences of different diagnostic marker gene subgroups were mainly concentrated in immune response and lipid metabolism, which indicated that changes in these two types of biological processes may play a key role in MMD.

### Correlation analysis among ACAN, FREM1, TOP2A and UCHL1 and immune infiltration

**Figure 9:**
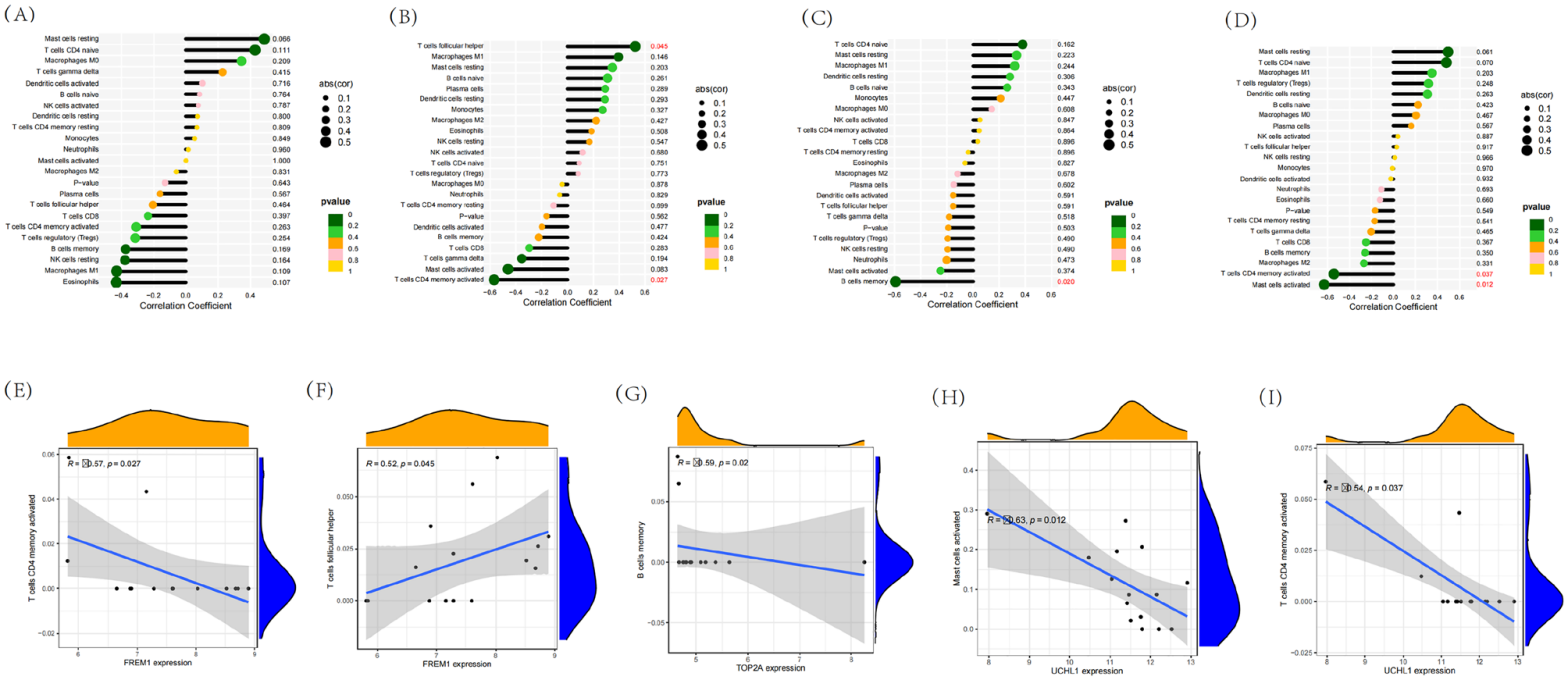
Correlation between diagnostic markers and infiltrating immune cells. (**A**–**D**) Correlation among hub genes and infiltrating immune cells. (**E**–**I**) The scatterplots showed the distribution of T cells follicular helper, T cells CD4 naive, B cells memory, T cells CD4 momory activated and mast cells activated count with p < 0.05 by Spearman’s rank correlation test. R > 0 indicated that the two were positively correlated, and vice versa.

**Figure 10:**
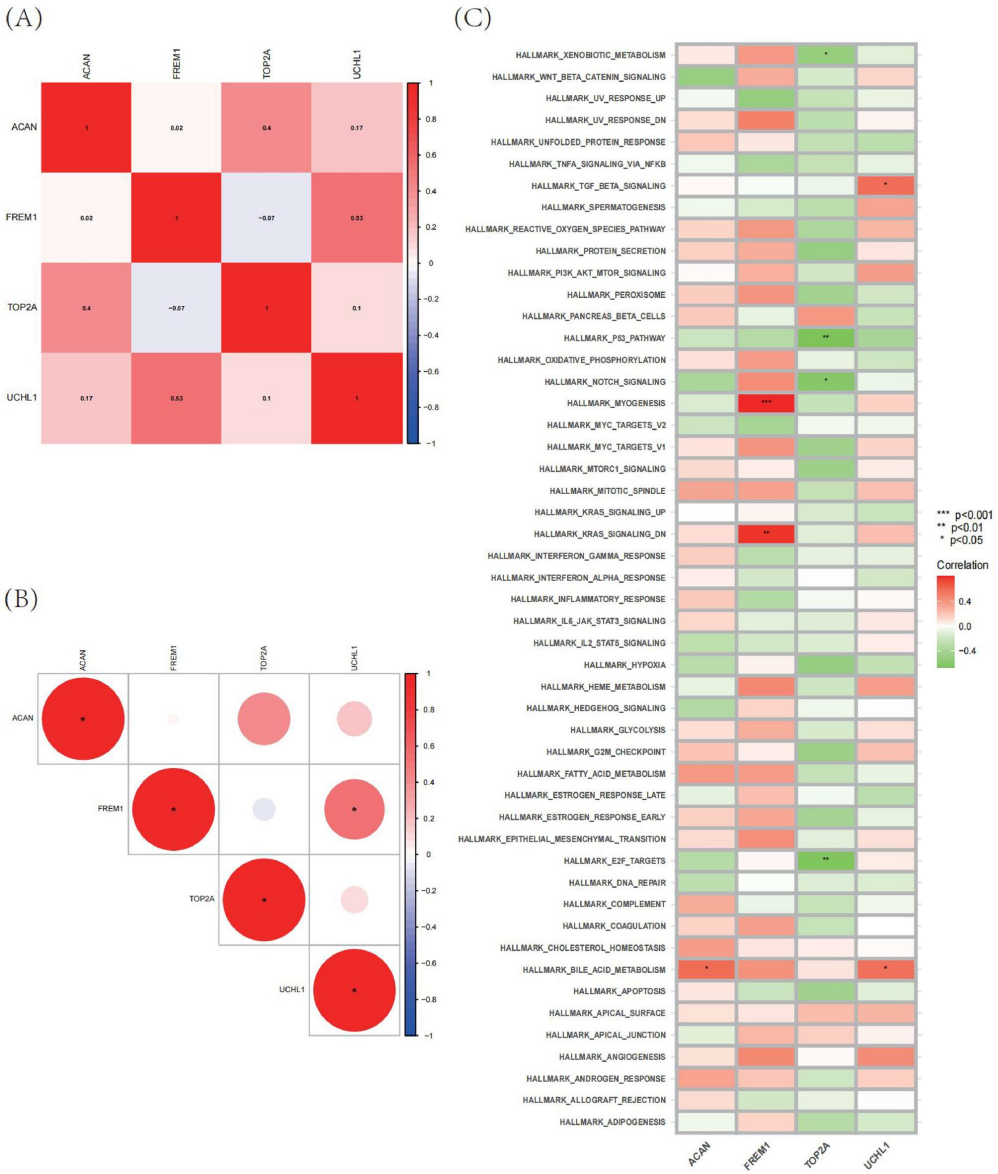
Visualization of immune cell infiltration and analysis of hallmark gene sets. (**A**,**B**) Correlation analysis of seven optimal feature genes in MMD samples. (**C**) Correlation analysis of the 50 hallmark gene sets with four optimal feature genes. Statistic tests: Wilcoxon rank-sum test (*P* < 0.2^#^; *P* < 0.05*; *P* < 0.01**; *P* < 0.001***; *ns* no significance).

We conducted Spearman correlation analysis to further clarify the correlation between key genes and various immune cell subsets. The results indicated that ACAN was positively correlated with mast cells resting (p = 0.066, considered marginally statistically significant) (Fig. 9A). FRM1 was positively correlated with T cells follicular helper (p = 0.045, r = 0.52), while it was negatively correlated with T cells CD4 naive (p = 0.027, r = − 0.57) (Fig. 9B,E,F). TOP2 was negatively correlated with B cells memory (p = 0.02, r = − 0.59) (Fig. 9C,G), UCHL1 was positively correlated with T cells CD4 momory activated (p = 0.037, r = − 0.54) and mast cells activated (p = 0.012, r = − 0.63) (Fig. 9D,H,I). Overall, T cells, B cells, and mast cells appear to be more closely associated with diagnostic marker genes in MMD and are more likely to play an important role in MMD. Gene correlations were also examined, as shown in Fig. 10A,B.

### Immune correlation analysis

**Figure 11:**
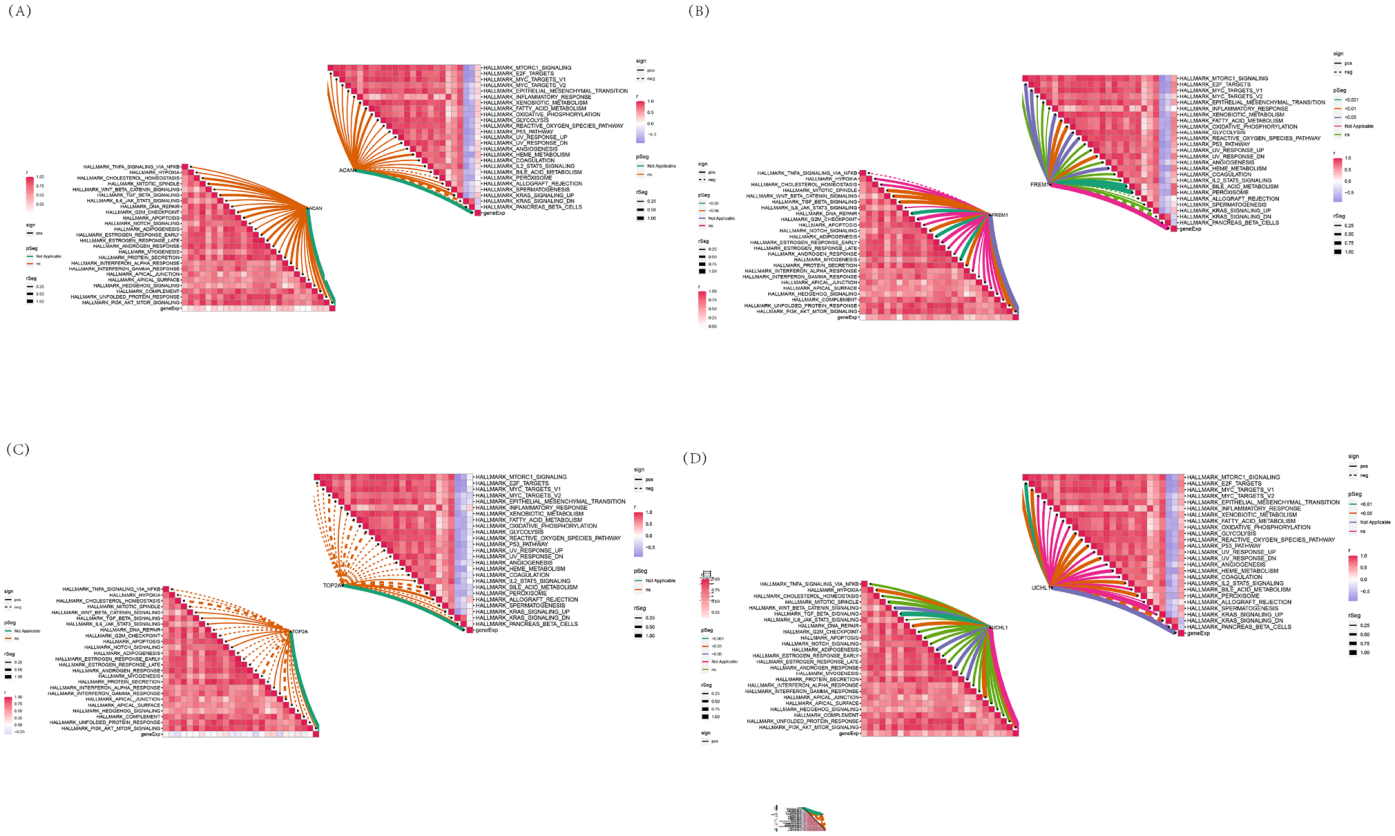
Correlation between characteristic genes and immunities. (**A**) Correlation between immune pathway and ACAN gene. (**B**) Correlation between immune pathway and FREM1 gene. (**C**) Correlation between immune pathway and TOP2A gene. (**D**) Correlation between immune pathway and UCHL1 gene.

To further assess the differences in the immune cell infiltration and hallmark gene sets between MMD and control samples, the CIBERSORT algorithm was employed. The results for differential immune cell infiltration are shown in Figs. 10C. The ssGSEA results of immune infiltration pathways involved and related to the correlation of shared hub genes are shown in the heatmap. ACAN was positively correlated with bile acid metabolism (Fig. 11A). Myogenesis and Kras signaling (DN) had strongly positively correlated with FREM1(Fig. 11B). E2F targets, NOTCH signaling, P53 pathway xenobiotic metabolism all had strongly negatively correlated with TOP2A (Fig. 11C). Metabolism, TGF beta signaling all had strongly positively correlated with UCHL1 (Fig. 11D). This indicates that these characteristic genes may regulate the immune process in the progress of MMD.

### Quantitative real-time PCR

**Figure 12:**
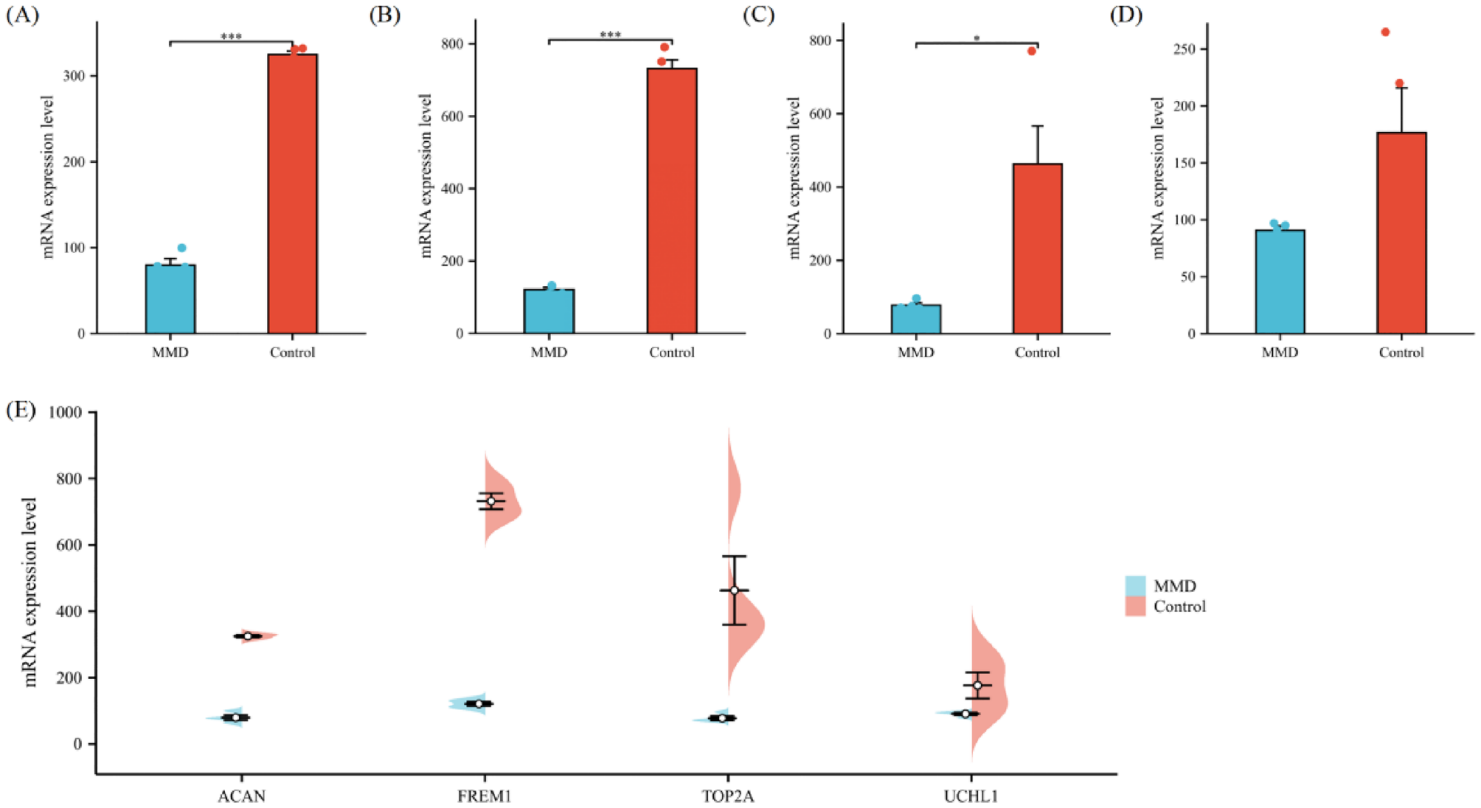
The qPCR of controls vs MMD groups. (**A**–**E**) mRNA level of ACAN, FREM1, TOP2A and UCHL1 in controls vs MMD groups. (p < 0.01). ** indicates P < 0.01.

To verify the expression of the 4 key genes in MMD, we obtained Peripheral venous blood samples from 4 patients with MMD and 4 normal subjects. The results of qPCR showed that the expression pattern of proliferation or differentiation genes was highly consistent with the bulk RNA-seq data. That is, compared with the control group, the expressions of ACAN, FREM1, TOP2A and UCHL1 in the experimental group were all decreased (Fig. 12A–E).

## Discussion

MMD is a disease in which the body attempts to compensate for this pathological feature by angiogenesis, forming smaller and weaker collateral vessels due to progressive stenosis of the cerebral arteries. However, these fragile vessels are more prone to bleeding, leading to adverse outcomes and even death. Unfortunately, although the clinical diagnosis and treatment of MMD has always been a difficult problem, the current understanding of the disease is still insufficient, and there is a lack of targets for early diagnosis and treatment. However, to data, many studies have proved the important role of immune system dysregulation in the process of MMD, which may be a good starting point for molecular research^24,25^.

The previous study by Jin et al. utilized bioinformatics methods to investigate the potential role of neutrophil-associated DEGs in MMD, and identified UNC13D as a promising candidate for characterizing neutrophil infiltration in MMD. However, we conducted a comprehensive analysis of all DEGs using three advanced machine learning algorithms to identify key genes, which were subsequently experimentally validated^26^. In this study, DEGs and WGCNA module genes were checked and combined, which screened out 153 candidate genes for further analysis. In addition, application of GO, KEGG, and DO enrichment assays to further investigate the potential functions and mechanisms of this module, DO analysis further uncovered that acute myocardial infarction (AMI) was significantly correlation with MMD, which is consistent with previous research. AMI is a critical symptom of coronary heart disease (CHD). Histopathological investigations of MMD-involved internal carotid arteries have shown that intimal fibroblast thickening, and smooth muscle cell (SMC) proliferation are responsible for arterial occlusion^27,28^. Furthermore, SMC proliferation is an integral part of the atherosclerotic mechanism of coronary artery disease, which is comparable to the histopathology of MMD. Besides, a previous study conducted to analyze explanted SMCs and myofibroblasts from patients carrying ACTA2 demonstrated increased proliferation of SMCs resulting in occlusive disease^29^. GO analysis showed that MMD was associated with heart-related regulation, neuronal release and other processes. Ikeda demonstrated that MMD is involved with the extra-cranial vessels as well as the intracranial vessels, and there are systemic etiologic factors, which cause intimal thickening in the systemic vessels^30^. Histopathologic studies of the involved internal carotid arteries in MMD showed fibrocellular thickening of the intima and proliferated smooth muscle cells (SMC) as the cause of the arterial occlusion^27,28^. The study conducted by Mika et al. unveiled a significant up-regulation of RNF213 mRNA, a susceptibility gene for MMD, in affected neurons as early as 6 h following transient focal cerebral ischemia and reperfusion. And the co-localization of Rnf213 mRNA expression with TUNEL-positive neurons suggests that the Rnf213 gene plays a role in cell survival and cell death in neural tissue under cerebral ischemia, which is an underlying pathology of MMD^31^. KEGG analysis showed that gastric cancer, breast cancer, hippo signaling pathway, hepatocellular carcinoma and wht signaling pathway were the most significant functional modules for enrichment. To prevent over-fitting, RF, LASSO, and SVM-RFE were selected to further screen for shared pivot genes, which were ACAN, FREM1, TOP2A and UCHL1.

Aggrecan, encoded by the ACAN gene, is a multi-module proteoglycan, accounting for 10% of cartilage. ACAN is vital in the morphogenesis of bone and cartilage, as well as several mutations have been found in short stature patients^32,33^. In patients with highly variable symptoms or syndrome phenotypes, at least 25 pathological ACAN mutations were found in nonsyndromic short stature^34^. However, the analysis of proteoglycan group confirmed that Acan exists in normal human aorta and also in aortic lesions of Acute type A aortic dissection (ATAAD) patients. A study revealed that ACAN plasma level is a reliable biomarker for detecting the presence of ATAAD. The marker can reliably detect ATAAD patients in a very sensitive way. Moreover, ACAN has a tight link with the occurrence and development of cancer. Vizeacoumar et al. showed that ACAN gene was significantly up regulated in all stages of cancer by comparison between normal gastric tissues and gastric tumors^35^. Recently, Vafaeie et al. illustrated that the diagnosed ACAN will serve as a new reference for the construction of a central gene-based prediction model for gastric cancer and provide new ideas for individualized treatment^36^. The same result also seen in LUAD^37^ and even endothelial dysfunction^38^. Interestingly, Jung et al. found that circulating endothelial progenitor cells isolated from peripheral blood of adult patients with MMD were dysfunctional^39^. All these studies have indicated that endothelial progenitor cells may be involved in the progressive occlusive injury of the internal carotid artery.

As for FREM1, a study illustrated that in cervical epithelial tissue, it may have a potential role in vaginal HIV-1 infection though enhancing mRNA expression of many inflammatory genes^40^. Another study found that many signaling pathways related to immune regulation were clustered in the high FREM1 expression group, such as inflammatory response, JAK-STAT signaling, cytokine-cytokine receptor interaction, and T cell receptor signaling^41^. These findings suggest that FREM1 may also be involved in the reconstruction and regulation of the immune microenvironment. However, the exact prognostic value of FREM1 in MMD patients still needs further investigation.

Multiple studies have revealed that UCHL1 is involved in some important human-related immune responses. Take for example, human papillomavirus induced UCHL1 expression in keratinocytes, which inhibited the secretion of macrophage inflammatory protein-3, type I interferon and interleukin-8 to promote the immune escape of human papillomavirus^42^. Gu et al. have proved that UCHL1 has a dual regulatory effect on the immunosuppressive ability of MSCs in inflammatory environment^43^. Similarly, MMD and these genes are closely related to immunity, and many autoimmune diseases are also related to moyamoya disease, which means that we need to more comprehensive and in-depth study the mechanism of the above genes involved in the formation of MMD.

Moreover, there is a lack of research in the MMD field regarding the TOP2A. TOP2A has been demonstrated to be involved in mechanisms of cancer formation and can be used as a biological predictor in a number of studies. Jain et al. found that the overexpression of TOP2A accelerated the progression of adrenocortical carcinoma^44^. TOP2A as a therapeutic target is also widely involved in clinical treatment, TOP2A change is a predictive marker of epirubicin sensitivity in clinical treatment^45^. TOP2A protein level can be used as a predictor of response of epirubicin to neoadjuvant therapy for breast cancer^46^.

Very interestingly, the 4 diagnostic marker genes and immune cell association analysis showed T cells, B cells, and mast cells may play an important role in MMD. The relationship between T cells and MMD was first pointed out in 1993. Studies have shown that the abnormally thickened vascular intima in MMD is mainly composed of smooth muscle cells and some macrophages and T cells^47^. In addition, a clinical study by Leihua Weng et al. also pointed out that the percentages of circulating Treg and Th17 cells in MMD patients were significantly higher than those in controls. In addition, it is interesting that their study also points to the important role of TGF-β in the progression of MMD^48^. This is consistent with the results obtained in our ssGSEA analysis. M Yamamoto et al. first pointed out that TGF-β1 is a potent enhancer of elastin expression in arterial SMC, and the expression of this gene is significantly increased in MMD patients^49^, while some other studies in recent years have pointed out that the polymorphism of TGF-β It is closely related to the progression of MMD in European races^50,51^, but research by Xiaomeng Wang et al. suggests that this polymorphism has no clear relationship with MMD in Chinese populations^52^. Similarly, the latest study by Shusuke Yamamoto et al. pointed out that the expression level of TGF-β1 in the cerebrospinal fluid of MMD patients was significantly increased, which may lead to the proliferation of fibroblasts in the arachnoid and their differentiation into myofibroblasts, thereby producing excess collagen, which in turn leads to the growth of malformed blood vessels in MMD. It is worth mentioning that changes in the TGF-β pathway show a high correlation with UCHL1, which has been verified in heart diseases and tumors^53,54^. Although this gene has not been studied in MMD, it is a potential intervention target. In addition, our immune infiltration analysis also suggested that there is a close relationship between B cells and mast cells and MMD, but unfortunately there is still a lack of such studies. Targets for therapeutic intervention. The GSEA analysis further enhanced the enrichment of relevant pathways. Firstly, MMPs play a pivotal role in vascular remodeling and angiogenesis, contributing to the development of collateral vessels in response to vessel narrowing and blockage in the brain^55,56^. Inflammation is a key player in the disease's development, with MMPs contributing to vascular inflammation and extracellular matrix degradation^57^. Additionally, certain MMPs are promising biomarkers, with elevated levels detected in individuals with moyamoya disease, indicating ongoing vascular remodeling and inflammation^58^. The study conducted by Miki Fujimura et al.^58^. utilized enzyme-linked immunosorbent assay to demonstrate that upregulated matrix metalloproteinase-9 (MMP-9) expression may contribute to the development of pathologic angiogenesis and/or destabilization of vascular structure, thereby potentially leading to bleeding in moyamoya disease. Muneaki et al.^59^. conducted immunohistochemical analysis of samples from patients with MMD and observed a significant accumulation of hyaluronic acid in the intimal thickening of occluding lesions associated with MMD. Hyaluronate synthase 2 was found to be highly expressed in endothelial progenitor cells exhibiting intimal thickening. It has been demonstrated that invading endothelial progenitor cells, aiming to repair endothelial damage, excessively produce hyaluronic acid within the intima, leading to vascular stenosis. Another aspect is the involvement of hyaluronic acid in the extracellular matrix (ECM). In moyamoya disease, ongoing vascular remodeling is a hallmark, potentially influenced by changes in the composition and distribution of hyaluronic acid, impacting the structural and mechanical properties of blood vessels^60,61^. Furthermore, hyaluronic acid interactions with specific receptors can contribute to inflammation and tissue damage^62,63^. Lastly, the disease's connection with ECM proteoglycans further underscores the role of vascular remodeling. These proteoglycans are integral to the structural and mechanical properties of blood vessel walls^64,65^. In inflammation, ECM proteoglycans can influence the inflammatory response, interacting with cytokines, growth factors, and immune cells. Maintaining extracellular matrix integrity is crucial for vascular health, and changes in proteoglycan content and distribution within blood vessel walls may affect the mechanical properties of these vessels^66,67^.

Our study is the first to incorporate machine learning to the identification of diagnostic markers for MMD, and the first to analyze the role of hub genes in MMD through GSEA. In addition, this study will help to identify effective targets for immunotherapy of MMD and promote the development of immunotherapy for MMD. At the same time, this work also outlined the map of MMD immune microenvironment, which provided a basis for the future research of MMD immune microenvironment.

## Conclusion

In conclusion, ACAN, FREM1, TOP2A and UCHL1 were established as diagnostic markers and potential immunotherapeutic targets for MMD by single cell, WGCNA, differential expression analysis and three machine learning methods. Immune infiltration analysis reveals a possible critical function of mast cells resting, T cells follicular helper, T cells CD4 naive, B cells memory, T cells CD4 momory activated and mast cells activated in the development of MMD, this could provide a novel insight into the pathogenesis and the joint treatment of MMD.

### Supplementary Information

Supplementary Figure S1.

Supplementary Table S1.

Supplementary Table S2.

Supplementary Table S3.

Supplementary Table S4.

Supplementary Table S5.

Supplementary Table S6.

## Data Availability

The original contributions presented in the study are included in the article/Supplementary Material; further inquiries can be directed to the corresponding authors. This sequencing dataset was obtained from GEO database (https://www.ncbi.nlm.nih.gov/geo/query/acc.cgi?acc=GSE141024, https://www.ncbi.nlm.nih.gov/geo/query/acc.cgi?acc=GSE157628).
