# Supplementary material for: Identification of diagnostic markers for moyamoya disease by combining bulk RNA-sequencing analysis and machine learning
Source: Sci Rep. 2024 Mar 11;14:5931. doi: 10.1038/s41598-024-56367-w (PMC10928210; doi:10.1038/s41598-024-56367-w)
Supplement: Supplementary file 1 — Supplementary Figure S1. [file 41598_2024_56367_MOESM1_ESM.docx]

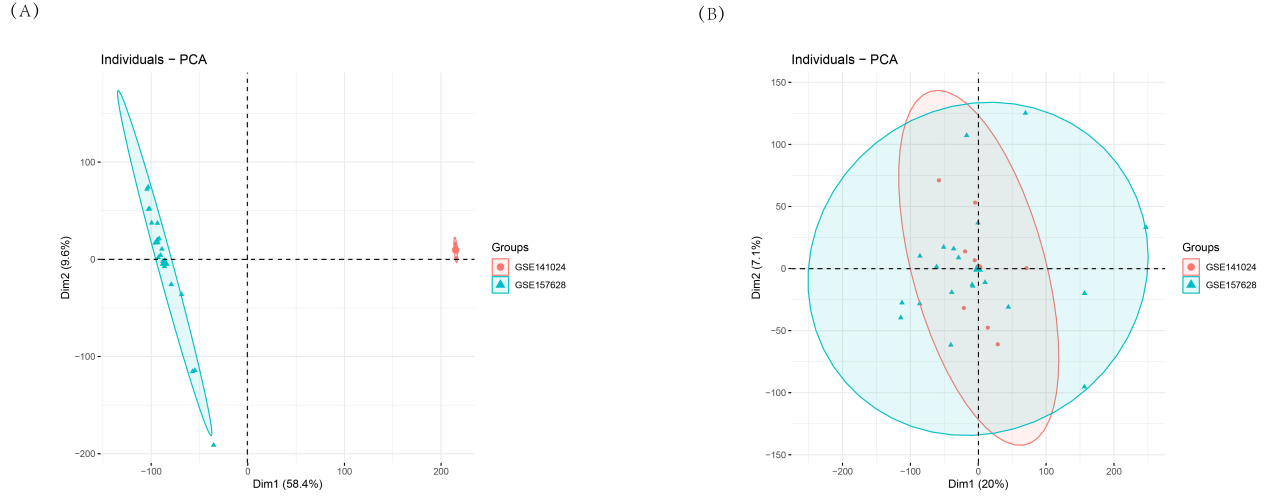


Figure S1. PCA performed on GSE141024, GSE157628. (A) PCA before removing batch effects. (B) PCA after removing batch effects.
